# Aging with HIV in the Shadows: How Older Asian Adults with HIV Navigate Stigma, Isolation, and Survival

**DOI:** 10.1093/geroni/igaf122.502

**Published:** 2025-12-31

**Authors:** Jen-Hao Chen, Cheng-Shi Shiu, Wei-Ti Chen

**Affiliations:** National Chengchi University, Taipei, Taipei, Taiwan (Republic of China); National Taiwan University, Taipei, Taipei, Taiwan (Republic of China); University of California, Los Angeles, Los Angeles, California, United States

## Abstract

This study examines how HIV-positive Asian immigrants navigate aging, disease management, and social marginalization in the United States, drawing on 34 in-depth interviews conducted in Los Angeles between 2018-2019. Our analysis reveals that the dual fear of being “found out” as HIV-positive and/or undocumented, combined with the need for resources, compels them to develop complex survival strategies while maintaining a double layer of secrecy in everyday interactions. The heightened stigmatization of HIV within Asian American communities leads many to hide their status from family and friends, withdraw from community life, and manage their illness and survival alone. In healthcare settings, withholding critical medical information to avoid discrimination and experiences of microaggressions is common. Unfortunately, such strategies limit access to social and healthcare support from communities and families that are often available to other older adults. Moreover, legal status and limited English proficiency frequently prevent them from leaving their ethnic communities, further restricting access to broader HIV resources and support networks and exacerbating social and healthcare precarity. Maintaining these strategies, however, creates heightened stress and takes a psychological toll. Consequently, these older adults age with HIV in the shadows, confined within Asian American communities, yet traditional support networks are often absent—or even sources of rejection. These findings challenge theoretical perspectives that frame family and communities as sources of support, highlighting their potential role as structural barriers to aging. The findings also underscore the need for interventions and policy reforms to address the compounded vulnerabilities of aging Asian immigrants with HIV.

